# Hypermethylation status of *DAPK*, *MGMT* and *RUNX3* in HPV negative oral and oropharyngeal squamous cell carcinoma

**DOI:** 10.1590/1678-4685-GMB-2019-0334

**Published:** 2020-08-21

**Authors:** Raquel Silva dos Reis, Jéssica Aflávio dos Santos, Priscila Marinho de Abreu, Raquel Spinassé Dettogni, Eldamária de Vargas Wolfgramm dos Santos, Elaine Stur, Lidiane Pignaton Agostini, Quézia Silva Anders, Lyvia Neves Rebello Alves, Isabella Bittencourt do Valle, Marília Arantes Lima, Evandro Duccini Souza, José Roberto Vasconcelos de Podestá, Sandra Ventorin von Zeidler, Melissa de Freitas Cordeiro-Silva, Iúri Drumond Louro

**Affiliations:** ^1^ Universidade Federal do Espírito Santo, Departamento de Ciências Biológicas, Núcleo de Genética Humana e Molecular, Vitória, ES, Brazil.; ^2^ Universidade Federal do Espírito Santo, Programa de Pós-Graduação em Biotecnologia, Vitória, ES, Brazil.; ^3^ Universidade Federal do Espírito Santo, Departamento de Patologia, Laboratório de Patologia Molecular, Vitória, ES, Brazil.; ^4^ Universidade Federal do Espírito Santo, Programa de Pós-Graduação em Ciências Fisiológicas, Vitória, ES, Brazil.; ^5^ Hospital Santa Rita de Cássia - SESA, Programa de Prevenção e Detecção Precoce do Câncer Bucal, Setor de Cirurgia de Cabeça e Pescoço, Vitória, ES, Brazil.

**Keywords:** tumor suppressor genes, methylation, HPV negative tumors, squamous cell carcinoma

## Abstract

Squamous cell carcinoma of the oral cavity and oropharynx is the sixth most common type of cancer in the world. During tumorigenesis, gene promoter hypermethylation is considered an important mechanism of transcription silencing of tumor suppressor genes, such as *DAPK*, *MGMT* and *RUNX3.* These genes participate in signaling pathways related to apoptosis, DNA repair and proliferation whose loss of expression is possibly associated with cancer development and progression. In order to investigate associations between hypermethylation and clinicopathological and prognostic parameters, promoter methylation was evaluated in 72 HPV negative oral and oropharyngeal tumors using methylation-specific PCR. Hypermethylation frequencies found for *DAPK*, *MGMT* and *RUNX3* were 38.88%, 19.44% and 1.38% respectively. Patients with *MGMT* hypermethylation had a better 2-year overall survival compared to patients without methylation. Being *MGMT* a repair gene for alkylating agents, it could be a biomarker of treatment response for patients who are candidates for cisplatin chemotherapy, predicting drug resistance. In view of the considerable levels of hypermethylation in cancer cells and, for *MGMT*, its prognostic relevance, *DAPK* and *MGMT* show potential as epigenetic markers, in a way that additional studies may test its viability and efficacy in clinical management.

## Introduction

Head and neck squamous cell carcinoma (HNSCC) is a heterogeneous group of epithelial tumors that describe approximately 90% of the malignant neoplasms occurring in the oral cavity, oropharynx, hypopharynx and larynx (Pai and Westra, 2009; Hussein *et al.*, 2017). Oral and oropharyngeal squamous cell carcinoma (OOSCC) have a global estimate of 300,373 and 142,387 new cases per year, respectively (Gupta *et al.*, 2016), being together the sixth most common cancer in the world (Morandi *et al.*, 2017).

A common etiological factor in HNSCC is prolonged and excessive alcohol and tobacco consumption which establish a synergistic, dose-dependent relationship between them (Polanska *et al.*, 2014). The infection by high-risk Human papillomavirus (HPV) has been particularly associated with oropharyngeal cancer (Lechner *et al.*, 2013; D'Souza and Saranath, 2015; Sailer *et al.*, 2017). HPV positive and HPV negative tumors have a distinct molecular profile, even in tumors with similar clinical parameters, leading to different prognostic expectations (Colacino *et al.*, 2013; Lechner *et al.*, 2013; Erhart *et al.*, 2016; Sailer *et al.*, 2017).

The OOSCC are very aggressive in their biologic behavior and result in a deforming and destructive disease, with frequent early lymph node metastases and potential for distant metastases over time – even after adequate local therapy (Miyazaki *et al.*, 2006; Byakodi *et al.*, 2012). These factors cause a significant worse prognosis and higher radio and chemotherapy morbidity (Morandi *et al.*, 2017). Mortality rates have remained unchanged (50% within five years after diagnosis) over the past 30 years (Morandi *et al.*, 2017) and survival rate of HNSCC is lower when compared to other cancers like breast, cervix and colorectal (Jemal *et al.*, 2008a, 2008b). Factors that contribute for this scenario are the failure in early diagnosis (two thirds are diagnosed in III–IV stages) and the lack of molecular markers that indicate tumor behavior and allow patient stratification for more personalized therapy (Mao *et al.*, 2004; Montebugnoli *et al.*, 2014; Morandi *et al.*, 2017).

Therefore, diagnoses in early stages and rigorous follow-up care have a significant effect on survival and outcome (Shield *et al.*, 2017). Currently, TNM staging system is the main parameter used for treatment decision and prognosis (Hermanek *et al.*, 1997). However, tumors with identical staging at same anatomical site can present distinct behavior (Pai and Westra, 2009). In this context, molecular markers for cancer detection and prognosis should be explored with the intent to improve screening accuracy (Pai and Westra, 2009; Carvalho *et al.*, 2011; Dahiya and Dhankhar, 2016).

DNA hypermethylation may be a suitable biomarker of tumor progression by allowing the prospection of malignant lesions and survival and prognostic associations (Taioli *et al.*, 2009; Koutsimpelas *et al.*, 2012; Castilho *et al.*, 2017). This molecular alteration consists of transcriptional silencing of promoter regions in tumor suppressor genes (TSGs) (Carvalho *et al.*, 2011; Castilho *et al.*, 2017).

CpG island hypermethylation in promoter region of TGSs as *death-associated protein kinase* (*DAPK*), *O* ^*6*^ *-methylguanine DNA methyltransferase* (*MGMT*) and *runt-related transcription factor 3* (*RUNX3*) have been consistently observed in many human cancers (Kito *et al.*, 2001; Raveh and Kimchi, 2001; Esteller and Herman, 2004; Subramaniam *et al.*, 2009; Asada *et al.*, 2013). These genes act in pathways of apoptosis, DNA repair and proliferative block, respectively, (Supic *et al.*, 2011) and their inactivation can favor oncogenesis and progression of oral tumors (Towle and Garnis, 2012; D'Souza and Saranath, 2015). In addition, all three genes were confirmed by an epigenome-wide methylation analysis using dysplastic and OSCC tissues (Towle *et al.*, 2013).

This study aimed to investigate the hypermethylation in *DAPK*, *MGMT* and *RUNX3* promoter regions and their association with clinicopathological features and the prognostic overall survival and disease-free survival in HPV-negative OOSCC.

## Subjects and Methods

### Ethical issues

This research was approved by the research ethics committee of the Integrated Center for Health Care – CIAS/Unimed Vitória (process number 318/2011). All patients were informed about the study and signed a written informed consent.

### Patients

The participants were recruited at the Head and Neck Surgery Section, Hospital Santa Rita de Cassia, located in Vitoria, Espírito Santo, Brazil, between 2011 and 2017. To prevent the existence of HPV status bias, the inclusion criteria were patients with conclusive diagnosis of OOSCC HPV DNA negative, who were not submitted to any antitumoral therapy. HPV negative status was confirmed by polymerase chain reaction (PCR) using GP5+/6+, MY09/11 and PGMY09/11 primer sets (Erhart *et al.*, 2016). Patients diagnosed with relapsed OOSCC were excluded as well as individuals with debilitating systemic conditions that limited their participation.

Clinical and pathological data (i.e., age, sex, tumour site, TNM stage, alcohol consumption and tobacco exposure) were obtained by interview and from the medical records. The tumor clinical stage was categorized as early (0, I and II) or advanced (III and IV) according to the TNM classification system (Wittekind *et al.*, 2014). Patients were considered non-smokers or non-alcoholics when they claimed never to have had the habit of alcohol or tobacco consumption. Volunteers were considered smokers or alcoholics when they smoked or had smoked on average one cigarette, cigar or pipe regularly for at least one year and ingested or had ingested alcoholic beverage (regularly/frequently) in life, respectively. Patients were followed up until 24 months or until death and outcomes were classified as alive, deceased and relapse.

### DNA extraction, quantification and sodium bisulfite modification

DNA was extracted from tumor samples stored at -80 °C originated of surgical resection. The used method was adapted from the phenol chloroform protocol from Goelz *et al.* (1985). DNA concentration was determined using *NanoDrop 2000* (Thermo Fisher Scientific, Delaware, USA). DNA integrity was evaluated by human β-globin gene PCR. Using the methylSEQr Bisulfite Conversion kit (Applied Biosystems, Foster City, California, USA), 300 ng of DNA from each sample were subjected to bisulfite conversion.

### Methylation Specific - Polymerase Chain Reaction (MS-PCR)

Each gene was tested with two different primers pairs: unmethylated (U) and methylated (M) both with previously described sequences and showed in Table 1. The primer pair U was specific for unmethylated alleles, rich in uracils, and the M primers were specific for methylated regions whose cytokines remain unchanged.

**Table 1:** Primer sequences, annealing temperature and size of MS-PCR amplicons used for *DAPK*, *MGMT* and *RUNX3* genes.

Gene	Allele	Sequence (5′- 3′)	AT^A^	Amplicon size in base pair	Reference
*DAPK*	U^B^	F^D^: GGAGGATAGTTGGATTGAGTTAATGTT	60 °C	106	Katzenellenbogen *et al.*, 1999
		R^E^: CAAATCCCTCCCAAACACCAA			
	M^C^	F^D^: GGATAGTCGGATGGAGTTAACGTC		98	
		R^E^: CCCTCCCAAACGCCGA			
*MGMT*	U^B^	F^D^: TTTGTGTTTTGATGTTTGTAGGTTTTTGT	55 °C	93	Esteller *et al.*, 1999
		R^E^: AACTCCACACTCTTCCAAAAACAAAACA			
	M^C^	F^D^: TTTCGACGTTCGTAGGTTTTCGC		81	
		R^E^: GCACTCTTCCGAAAACGAAACG			
*RUNX3*	U^B^	F^D^: AAGTGGGAAAGTAGAAGTGGTG	62 °C	125	Kim *et al.*, 2004
		R^E^: CCAAACAAACTACAAACAACCA			
	M^C^	F^D^: TATTCGTTAGGGTTCGTTCGT	61 °C	119	
		R^E^: AAACAACCACGAAAAACGAC			

Abbreviations: A: annealing temperature; B: unmethylated; C: methylated; D: forward primer; E: reverse primer.

Normal lymphocyte DNA methylated *in vitro* by the CpG Methyltransferase enzyme (M.SssI) (Thermo Fisher Scientific) was used as reference control of hypermethylated sequence*.* This sample was used as positive amplification control for M primers and negative control for U primers. DNA from normal lymphocytes was used as reference for unmethylated sequences, serving as positive control for U primers and negative control for M primers. Both controls have been modified by bisulfite as described.

The PCR reaction mixture consisted of 2 μl of modified DNA, 0.072 mM of each dinucleotide triphosphate (Invitrogen/Life Technologies, California, USA), 1X of PCR buffer (50 mM KCl, 20 mM Tris-HCl pH 8.4) of the Platinum Taq DNA Polymerase (Invitrogen), 0.54 mM of MgCl_2_ (Invitrogen), 0.9 U of Platinum Taq DNA Polymerase (Invitrogen) and 0.6 μM of each primer (Invitrogen).

Fragments were amplified in a Veriti 96-Well Thermal Cycler (Applied Biosystems) under the following conditions: initial denaturation at 95 °C for 2 min; 35-38 cycles consisting of denaturation at 95 °C for 30 s; annealing at a specific temperature (AT) for 30 sec and extension 72 °C for 30 s; final extension of 10 min at 72 ° C. The MS-PCR conditions are shown in Table 1. The MS-PCR products were subjected to electrophoresis on 7% polyacrylamide gel stained with silver nitrate.

### Statistical analysis

Statistical analysis was performed with SPSS Statistics v20.0 (SPSS Incorporation, Chicago). The association between variables was tested by Chi-Square test or Fisher's exact test. All variables with a p-value <0.25 in association tests were subjected to multiple logistic regression analysis. The overall survival and disease-free survival were estimated and compared using the Kaplan-Meier method and log-rank tests, respectively. Multivariate Cox regression analysis was performed to verify if there was any association between the variables when evaluated together by the same model. Associations were considered significant when p < 0.05.

## Results

### Patients

Seventy-two patients were included in the study. The mean age was 57.25, ranging from 31 to 84 years old. Alcoholism was reported by 60.86% (42/69) and smoking was assumed by 62.31% (43/69). Those who claimed not to use any of these substances amounted to 21.73% (15/69). Most patients (69.44%; 50/72) were at advanced stages of the disease (III and IV), with tumors T3 and T4 accounting for 55.55% (40/72) of samples. One patient had distant metastasis. Follow-up data were obtained for 81.94% (59/72) of the patients. During this period, 13.56% (8/59) of the patients developed recurrence, and 35.59% (21/59) died. The clinicopathological description of patients is shown in Table 2.

**Table 2:** Clinicopathological features and their distribution according *DAPK* and *MGMT* hypermethylation.

Clinical Features^A^		**Total (n)**	*DAPK*					*MGMT*				
			**Hypermethylated**		**Non-hypermethylated**		**p-value** ^B^	**Hypermethylated**		**Non-hypermethylated**		**p-value** ^B^
		**72**	**n**	**(%)**	**n**	**(%)**		**n**	**(%)**	**n**	**(%)**	
Sex	Male	58	25	43.1	33	56.9	0.223	11	19.0	47	81.0	1.000
	Female	13	3	23.1	10	76.9		2	15.4	11	84.6	
Age (years)	< 57.5	36	16	44.4	20	55.6	0.332	7	19.4	29	80.6	1.000
		≥ 57.5	35	12	34.3	23	65.7		6	17.1	29	82.9	
Anatomical Site	Oral Cavity	56	22	39.3	34	60.7	1.000	10	17.9	46	82.1	0.169
	Oropharynx	16	6	37.5	10	62.5		4	25.0	12	75.0	
TNM stage	Tis/T1/T2	22	10	45.5	12	54.5	0.600	7	31.8	15	68.2	0.107
	T3/T4	50	18	36.0	32	64.0		7	14.0	43	86.0	
Tumor Size	Tis/T1/T2	32	16	50.0	16	50.0	0.095	8	25.0	24	75.0	0.373
	T3/T4	40	12	30.0	28	70.0		6	15.0	34	85.0	
Lymph node Involvement	Yes	33	14	42.4	19	57.6	0.632	4	12.1	29	87.9	0.232
	No	39	14	35.9	25	64.1		10	25.6	29	74.4	
Chemotherapic treatment	Cisplatin	21	10	47.6	11	52.4	0.593	2	9.5	19	90.5	0.422
	Others	4	3	75.0	1	25.0		1	25.0	3	75.0	
Smoking	Yes	43	17	39.5	26	60.5	1.000	9	20.9	34	79.1	1.000
	No	26	10	38.5	16	61.5		3	11.5	23	88.5	
Alcohol consumption	Yes	42	18	42.9	24	57.1	0.460	9	21.4	33	78.6	0.342
	No	27	9	33.3	18	66.7		3	11.1	24	88.9	
Smoking and Alcohol consumption	Yes	31	12	38.7	19	61.3	0.805	7	22.6	24	77.4	0.352
	No	38	14	36.8	24	63.2		5	13.2	33	86.8	

Abbreviations: A: Some variables have missing data; B: Chi-Square test or Fisher's exact test.

### Methylation profile of *DAPK*, *MGMT* and *RUNX3*

Hypermethylation was found in 38.88% (28/72) of the tumors for *DAPK* and 19.44% (14/72) for *MGMT.* Hypermethylation in at least one of the genes was found in 48.61% (35/72) of the cases. Simultaneous hypermethylation for *DAPK* and *MGMT* reached 9.72% (7/72). Hypermethylation at *RUNX3* was observed in one sample (1.38%) which presented all genes hypermethylated.

### Clinicopathological features and hypermethylation of *DAPK* and *MGMT* genes

The patients' clinicopathological description according to hypermethylation of *DAPK* and *MGMT* genes is summarized in Table 2. There were no statistically significant results in the association tests between hypermethylation results and the following clinicopathological data: age, sex, smoking and alcohol addiction anatomical site, tumor stage, tumor size, lymph node involvement and chemotherapic treatment. Variables that presented p <0.25 in association analyses were used in a multiple logistic regression analysis. As shown in Table 3, we observed that individuals with initial tumor size (Tis, T1 and T2) were 3.35 times (95% CI 1.093 – 10.329) more likely to have methylation of the *DAPK* gene than those who did not. The Hosmer-Lemersshow test demonstrated that the regression model was adequate (p = 0.384). None of the other independent variables analyzed were shown to predict gene methylation events. Analyses were not performed for *RUNX3*.

**Table 3:** Multiple logistic regression of clinical features and *DAPK* and *MGMT* hypermethylation.

Clinical Features^A^		*DAPK*		
		OR (Adjusted)^B^	95% CI^C^	p-value^D^
Sex	Male	1^E^	0.588 - 10.607	0.215
	Female	2.497		
Tumor Size	Tis/T1/T2	3.359	1.093 - 10.329	**0.034**
	T3/T4	1 ^E^		
Lymph node Involvement	Yes	1.874	0.608 - 5.779	0.274
	No	1 ^E^		
Clinical Features		*MGMT*		
		OR (Adjusted)^B^	95% CI^C^	p-value^D^
Anatomical Site	Oral Cavity	1 ^E^	0.563 - 41.066	0.151
	Oropharynx	4.809		
Tumor Size	Tis/T1/T2	1.663	0.441 - 6.275	0.453
	T3/T4	1 ^E^		
Lymph node Involvement	Yes	0.503	0.123 - 2.051	0.338
	No	1 ^E^		

Abbreviations: A: Some variables have missing data; B: Odds Ratio; C: Confidence Interval; D: Multiple logistic regression (Adjusted to all variables); E: Reference category.

### Overall survival (OS) and Disease free survival (DFS)

In OS and DFS evaluation, the 2-year survival was analyzed according to *DAPK* and *MGMT* methylation status, clinical staging and tumor size (T) and lymph node metastasis (N).

**Figure 1:**
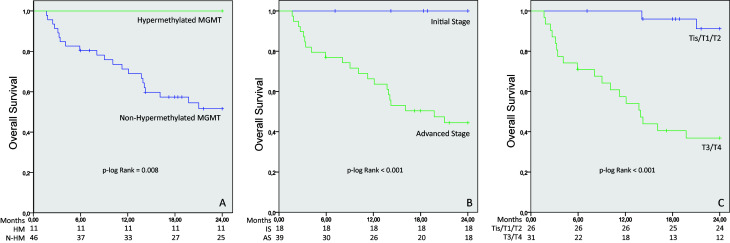
Kaplan Meier estimates of overall survival among patients with oral and oropharyngeal squamous cell carcinoma according to MGMT hypermethylation, Tumor Stage and Tumor Size. Abbreviations: A: MGMT hypermethylation; B: Tumor Stage; C: Tumor Size; HM: Hypermethylated; N-HM: Non-Hypermethylated; IS: Initial Stage; AS - Advanced Stage

Abbreviations: A: *MGMT* hypermethylation; B: Tumor Stage; C: Tumor Size; HM: Hypermethylated; N-HM: Non-Hypermethylated; IS: Initial Stage; AS - Advanced Stage.

As shown in Figure 1, patients with hypermethylation of *MGMT* gene had better overall survival (p=0.008) than patients without. All patients with hypermethylation of *MGMT* remained alive until the end of the follow-up, while approximately 50% of patients without hypermethylation remained alive until the end of the follow-up, as seen in Figure 1. Patients in early staging (0, I and II; p < 0.001) and with smaller tumors (T1 and T2; p < 0.001) had better survival. All patients in early staging remained alive until the end of the follow-up while more than 60% of patients with larger tumors died during follow-up.

The multivariate Cox regression analysis was performed in order to confirm whether the hypermethylation of the evaluated genes and the prognostic factors (tumor size and lymph node metastasis) showed any association when evaluated together in the same model. As observed in Table 4, the multivariate regression showed that tumor size showed an association with survival (p=0.001). Therefore, we can infer that patients with advanced tumors have 13.42 times the risk of death.

**Table 4:** Multivariate Cox regression analysis of prognostic features and *DAPK* and *MGMT* hypermethylation.

Variables	HR^A^	95% CI for HR^B^	p-value^C^
DAPK	0.71	0.28 - 1.83	0.476
MGMT	6.60E+05	1.44E-259 - 3.02E+270	0.966
Tumor Size	13.42	2.88 - 62.52	**0.001**
Lymph node Involvement	1.01	0.42 - 2.45	0.984

Abbreviations: A: Hazard Ratio; B: Confidence Interval; C: Multivariate cox regression.

## Discussion

Hypermethylation of CpG islands in genes related to cancer has been considered an important event in OOSCC evolution. We have evaluated the methylation status of the *DAPK*, *MGMT* and *RUNX3* genes in 72 OOSCC HPV negative tumors. Among these genes, hypermethylation in the *MGMT* gene correlates with better overall survival.

Promoter hypermethylation rates found in our study fit on intervals reported in the HNSCC literature for *DAPK* (12 – 71.69%) (Li *et al.*, 2013; Noorlag *et al.*, 2014) and *MGMT* (18.1 – 58.67%) (Zuo *et al.*, 2004; Strzelczyk *et al.*, 2018), but not for *RUNX3* (15 – 70%) (Gao *et al.*, 2009; Zhang *et al.*, 2013).

Multiple logistic regression analysis alone demonstrated a significant association between individuals with initial tumor size and methylation of the *DAPK* gene; *MGMT* hypermethylation data were not associated with clinicopathological parameters in our study. In the studies of Martone *et al.* (2007), Steinmann *et al.* (2009), Rettori *et al.* (2013) and Misawa *et al.* (2016) associations were not observed between the status of the *MGMT* and *DAPK* genes and tumoral and clinical parameters.

*MGMT* encodes an adduct repair enzyme of O^6^-methylguanine generated by the interaction of DNA with alkylating agents present, for example, in cigarette smoke (Christmann and Kaina, 2012). The *MGMT* promoter methylation can reduce the protein expression having therefore oncogenic potential (Cai *et al.*, 2016). Although our report is not the first to point out the prognostic value of *MGMT* for HNSCC patients, there is no consensus among studies previously published. Taioli *et al.* (2009) and Zuo *et al.* (2004) found an inverse relationship between hypermethylation and OS in samples of OOSCC and HNSCC, respectively. Whereas Dikshit *et al.* (2007), studying laryngeal and hypopharyngeal tumors, and Strzelczyk *et al.* (2018), working with oral cavity cancer, found no association with survival. Despite differences in etiology and behavior, our results of improved OS associated to *MGMT* hypermethylation better fit in the conclusions of Chen *et al.* (2015). In their cohort of cisplatin-treated nasopharyngeal cancer patients, worse OS was observed for those with high *MGMT* expression associated with absence of hypermethylation.

Cisplatin and the others platinum-containing anti-cancer drugs are widely used in the treatment of locally advanced HNSCC tumors (Schmitz *et al.*, 2014). It acts as an alkylating agent leading to formation of platinum-DNA adducts blocking cell cycle and resulting in cancer cell apoptosis (Dasari and Tchounwou, 2014). Although cisplatin is not an O^6^-alkylguanine alkylating agent, its DNA damage is actively removed by *MGMT* repair protein, as demonstrated by Chen *et al.* (2015). Thus, *MGMT* hypermethylation, and its consequent loss of expression, could be a prognostic biomarker for HNSCC patients candidates for cisplatin chemotherapy, predicting the chances of drug resistance. Since most patients begin treatment at later stages, the therapeutic approach may be more effective when oriented by the molecular tumor profile. It has been shown that *MGMT* promoter methylation is a good prognostic factor for patients with glioma treated with temozolomide, another alkylating drug (Pandith *et al.*, 2018). In our study, however, there was no statistically significant relationship between the use of cisplatin and methylation of *MGMT* gene. This may be due to the small number of samples. Furthermore, the disagreement about survival results among the studies already published may be related to anatomical site differences, HPV status and patient's clinical stage.

In relation to TSG *DAPK*, preview studies reveal that its hypermethylation is a common phenomenon in HNSCC (Kulkarni and Saranath, 2004; Li *et al.*, 2013; Misawa *et al.*, 2016). It is known that *DAPK*, which is a mediator of cell death of interferon-gamma (INF-γ)-induced apoptosis (Cai *et al.*, 2017), is essential for activation of various cell death mechanisms, caspase dependent or not, and the p19ARF/p53 signaling pathway, a classic pathway in cell cycle control (Bialik and Kimchi, 2004). Since death pathways abolition is critical for tumor growth, *DAPK* hypermethylation may be suggestive of a higher malignant potential. This was correlated with lymph node involvement in studies of Sanchez-Cespedes *et al.* (2000) and Wong *et al.* (2011). Considering that epigenetic regulation is an early event in oral carcinogenesis (D'Souza and Saranath, 2015), its detection in normal tissue adjacent to the tumor (Kulkarni and Saranath, 2004; Wong *et al.*, 2011), pre-cancerous lesions (Liu *et al.*, 2012), and surgical margins (Martone *et al.*, 2007) could contribute to the monitoring of tumor progression and clinical management in view of both over- and under treatment has an impact on patient morbidity (Shridhar *et al.*, 2016). In this study, the multiple regression analysis demonstrated that tumors with sizes considered initial (Tis, T1 and T2), had a greater chance of being *DAPK* methylated. The detection of early epigenetic events indicative of malignant potential can favor the diagnosis in early stages (0, I and II) and in the discovery of still small lesions (T1 e T2) (D'Souza and Saranath, 2015). As evidenced in this and other studies, it is known that these last two factors are indicative of good prognosis.

The low rate of hypermethylation found for *RUNX3* in this study (1.38%) questions its importance in HNSCC genesis and development. *RUNX3* is commonly presented as an effector of the transforming growth factor beta (TGF-β) pathway known for its effects of inhibiting growth and promoting apoptosis (Bae and Choi, 2004). Recently, studies with HNSCC have suggested an oncogenic role of *RUNX3*. Tsunematsu *et al.* (2009) and Kudo *et al.* (2011) argue that in healthy oral mucosa this gene would be epigenetically silenced, since its expression would only be required during embryonic development. During carcinogenesis, its oncogenic action would be triggered by the demethylation of its CpG island, favoring cell growth and inhibition of apoptosis. It is possible that this gene has a dual performance in the development of the HNSCC, which could help in understanding the apparent inconsistencies between the results of this research with others in the literature. According to Lebrun (2012), the TGF-β pathway may exhibit tumor suppressor action in the early stages and promotes invasion and metastasis in later stages. It is worth noting that even in studies in which hypermethylation was detected (Supic *et al.*, 2011; Cordeiro-Silva *et al.*, 2012), a relevant portion of the samples (75% and 83%, respectively) did not present hypermethylation, suggesting that *RUNX3* may be expressed.

*DAPK*, *MGMT* and *RUNX3* are TSGs whose hypermethylation has been reported as an important event in HNSCC. In the present study with HPV-negative oral and oropharynx tumors, only *DAPK* and *MGMT* showed consistent evidence of their potential as epigenetic markers, with considerable levels of hypermethylation in cancer cells and, for *MGMT*, prognostic relevance. These results could contribute to improve strategies in early diagnosis and follow-up guided by evaluation of gene methylation patterns. However, it is important to highlight the need of further studies and clinical trials to define, for each subtype of HNSCC tumor, the actual diagnostic and predictive value of *DAPK* and *MGMT* as well as its viability and efficacy in clinical management.
